# Visualization and Semantic Labeling of Mood States Based on Time-Series Features of Eye Gaze and Facial Expressions by Unsupervised Learning

**DOI:** 10.3390/healthcare10081493

**Published:** 2022-08-08

**Authors:** Hirokazu Madokoro, Stephanie Nix, Kazuhito Sato

**Affiliations:** 1 Faculty of Software and Information Science, Iwate Prefectural University, Takizawa 020-0693, Japan; 2 Faculty of Systems Science and Technology, Akita Prefectural University, Akita 015-0055, Japan

**Keywords:** facial expressions, human communication, mental health, saccades, self-organizing maps, U-Matrix

## Abstract

This study is intended to develop a stress measurement and visualization system for stress management in terms of simplicity and reliability. We present a classification and visualization method of mood states based on unsupervised machine learning (ML) algorithms. Our proposed method attempts to examine the relation between mood states and extracted categories in human communication from facial expressions, gaze distribution area and density, and rapid eye movements, defined as saccades. Using a psychological check sheet and a communication video with an interlocutor, an original benchmark dataset was obtained from 20 subjects (10 male, 10 female) in their 20s for four or eight weeks at weekly intervals. We used a Profile of Mood States Second edition (POMS2) psychological check sheet to extract total mood disturbance (TMD) and friendliness (*F*). These two indicators were classified into five categories using self-organizing maps (SOM) and U-Matrix. The relation between gaze and facial expressions was analyzed from the extracted five categories. Data from subjects in the positive categories were found to have a positive correlation with the concentrated distributions of gaze and saccades. Regarding facial expressions, the subjects showed a constant expression time of intentional smiles. By contrast, subjects in negative categories experienced a time difference in intentional smiles. Moreover, three comparative experiment results demonstrated that the feature addition of gaze and facial expressions to TMD and *F* clarified category boundaries obtained from U-Matrix. We verify that the use of SOM and its two variants is the best combination for the visualization of mood states.

## 1. Introduction

The advanced progress of information technologies in our society provides usefulness, accessibility, and convenience to our daily lives. Particularly with the COVID-19 pandemic, the need for remote work, online meetings, and online learning has rapidly spread around the world [1,2,3,4,5]. By virtue of modern widespread internet technology, huge amounts of digital data, including big data [6], are circulating rapidly in real-time around the world, not only with global information provided as news articles from mass media but also with local information posted from bloggers and community information exchanged using social networking services (SNS) [7]. Simultaneously, regarding negative aspects, various difficulties have arisen, such as invasion of privacy, lack of computer literacy, unfounded rumors, and fake news [8]. The emergence of deepfakes [9] that can produce hyper-realistic videos using deep learning (DL) networks [10] accelerates this issue [11,12,13,14].

In addition, industrial products and computer interfaces that are unfamiliar to people inhibit satisfactory living and social activities to everyone’s desire that they be convenient. Users who try to force themselves to fit in with many situations might feel uncomfortable, frustrated, and stressed. Actually, the dominant industrial structure in modern society has changed from manufacturing industries in the last century to information industries, which process large amounts of data as digital codes in real-time [15]. Computers, tablets, and smartphones play important roles as powerful tools in marketing activities and our current digitalized society [16]. Particularly, DL technologies and applications have boosted this progress, especially during its major transition in 2012 [17]. However, these digital devices have induced numerous instances of confusion in human communication [18]. The use of irrational, difficult, and complex hardware and software often induces stress factors. Therefore, numerous people spend their daily lives and businesses coping with stressors of various types that are attributable to these influences and realities.

Stress occurs as a vital response of the brain and body to cope with stressors [19]. Individual variations exist in stress response, tolerance, and emotional patterns. Therefore, the magnitude of stress varies even among people along with individual differences [20], even in similar environments, conditions, situations, circumstances, and contexts. Usually, in a healthy condition, the brain and body respond appropriately to emphasize maintenance of physical and mental balance. However, excessive stress induces abnormalities in the mind and body. In the worst case, we are adversely affected by mental illnesses such as depression, psychosomatic disorders, and neurosis [21]. Transformation of the industrial structure and business style decreases physical illness and increases mental illness. Particularly, those who work in the service industry encounter widely various stressors in their daily work.

In Japan, a stress check program was introduced in 2014 with a revision of the Industrial Safety and Health Act [22]. Since 2015, stress check tests have been imposed as an obligation for organizations that have more than 50 employees. However, organizations employing fewer than 50 workers are simply asked to make an effort to do so. Stress check tests are conducted at one-year intervals by a medical doctor or by a public health nurse. This frequency is unsuitable for the early detection of stress accumulated in daily lives. Therefore, recognizing mild discomfort as a sign of stress plays an important role in stress management. Moreover, tools, methods, and systems that can measure stress simply, easily, readily, and frequently over a long period are expected to be necessary for modern high-stress societies. This study is intended to develop a stress measurement and visualization system for stress management in terms of simplicity and reliability. Particularly, we prototyped a mental health visualizing framework based on machine learning (ML) algorithms as a software tool that can feed back analytical signals of stress measurements [23].

Most existing studies have specifically examined subjective responses under transient stress [24]. Long-term periodic stress observations are related closely to slight changes in mental conditions. However, this study was conducted to collect an original dataset related to chronic stress obtained from university students. Usually, university students feel burdened with their daily routines and schoolwork, which might be expected to include attending lectures, club activities, working as a teaching or research assistant as a part-time job, writing reports, and research on a graduation thesis [25]. This study specifically examined the correlation between chronic stress and biometric signals obtained as physical responses. We obtained an original dataset related to stress responses from psychological and behavioral indexes. Particularly, we obtained two psychological scores related to mood states and time-series images that included facial expressions, gaze distributed patterns, and the number of saccades.

This study is intended to visualize features related to mental health using ML algorithms. We hypothesized that emotional changes resulting from different chronic stress conditions affect gaze movements and facial expression changes. Our earlier feasible research results [23] demonstrated that the degree of gaze concentration tended to be related to the psychological state. This study was conducted to verify the relation between gaze movements, including saccades, and chronic stress in daily life without using external stimuli. Feature signals of gazing and facial expressions are obtainable using a nonrestricted measurement approach. Therefore, burdens for subjects are lower than that of restricted or contact measurement methods. Experimentally obtained results obtained from 20 subjects demonstrated a tendency of feature patterns visualized on category maps for analyzing stress responses in each subject.

In this study, we used self-organizing maps (SOMs) [26] for unsupervised clustering and data visualization [27]. Because of containing both properties, SOMs have been widely used in various and numerous studies in the era of mainstream DL algorithms. Compared with DL algorithms that require a vast of data, one important advantage for SOMs is to conduct steady learning with relatively lower computational resources and calculation costs. Recent research examples of clustering, visualization, recognition, classification, and analyses using SOMs comprise medical system applications [28,29,30,31,32], social infrastructure maintenance [33,34,35,36,37,38], consumer products and services [39,40,41,42,43], food and smart farming [44,45,46], and recycling and environmental applications [47,48,49,50,51,52,53]. We employed SOMs and their variants for the task of classification and visualization of mood states.

This paper is structured as follows. Section 2 briefly reviews state-of-the-art stress measurement systems and methods, especially non-invasive and non-contact approaches. Subsequently, Section 3 and Section 4, respectively, present our original benchmark dataset and our proposed method consisting of four ML algorithms. Experiment results of classification and visualization of mood states related to gaze features and facial expressions are presented in Section 5. Finally, Section 6 presents conclusions and highlights future work.

## 2. Related Studies

Studies of mental stress have been undertaken from two perspectives: stressors caused by mental or physical stimulus and psychosomatic responses to stressors. However, it is still a challenging research task to quantify stressors and psychosomatic responses, especially in differences in feelings among individuals. Inaba et al. [54] specifically examined psychological differences between couples before marriage, which is socially positioned as a seemingly good life change, to analyze individuals in terms of their reactions to stressors arising from similar causes. They verified changes that occurred from stress factors: not only excessive quotas and long working hours but also life-changing events such as advancement to higher education, employment, marriage, and job promotion.

**Figure 1:**
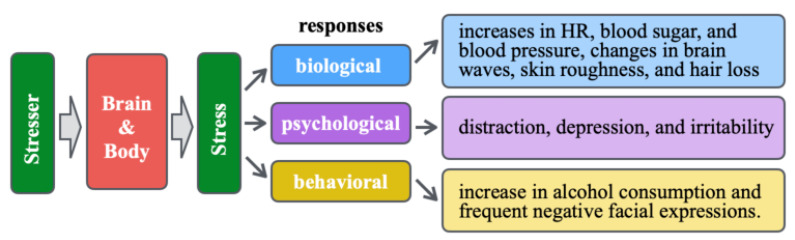
Stress types and representative responses.

As depicted in Figure 1, stress elicits biological, psychological, and behavioral responses after being processed in the brain. Representative biological responses comprise increases in heart rate (HR), blood sugar and blood pressure, brain wave changes, skin roughness, and hair loss. Representative psychological responses include distraction, depression, and irritability. Representative behavioral responses comprise an increase in alcohol consumption and frequent negative facial expressions. In a modern, stressful society, few indexes or tools are available to ease the assessment of the quantity and quality of stress, including a person’s mental state [55].

Although stress is a subjective phenomenon, measurement and assessment are performed objectively. Objective information is obtainable not only from biological signals, such as blood, saliva, and hair, but also from physiological signals, such as blood pressure, pulse, HR variability, and blinking. Representative approaches include assessment using stress test sheets [56] and assessment from responses using an HR sensor [57] or a salivary amylase test [58]. Takatsu et al. [59] and Matsumoto et al. [60] verified that fluctuations in HR correlate with stress responses. By contrast, a salivary amylase test, which requires a special measurement instrument, is difficult to measure frequently and casually. Approaches that specifically examine gaze and facial expressions as behavioral information are being researched actively [61,62,63,64]. Face image-based approaches are expected to be developed or incorporated into applications [65,66,67] to assess mental health from images obtained using a camera on a smartphone, including smart glasses and a smart mirror.

Stress can be classified roughly as either chronic stress or transient stress [68]. Chronic stress occurs from stressors over a long period. Transient stress occurs in situations characterized by a concentration of strain that results from temporary factors. Although most earlier studies targeted transient stress, our study specifically examines chronic stress because daily stress changes over a long period. Particularly, our study is designed to classify mood states for detecting early disorders through visualization of the relation between physical reactions and mental health using ML algorithms. Our target measurement signals are gaze and facial expressions that can be measured using a non-contact and non-constraint approach. Compared to other methods, this measurement approach can avoid causing stress. Moreover, we set up an experiment environment considering human communication.

### 2.1. Gaze and Saccades

The human gaze enables monitoring, representation, and coordination functions. The monitoring function collects perceptual information of a target and its surrounding environmental information as context. The representation function conveys intentions and emotions to surrounding people. The coordination function gives and receives statements in conversation. These functions play important roles in human communication and social interaction. Vision is an important and complex perception: through vision, almost everyone gets tremendous amounts of information [69]. Moreover, rapid eye movements, termed saccades, occur for visual confirmation. Saccades act to capture an object in the central fovea of the retina [70].

As a study of saccades, Mizushina et al. [71] emphasized specifically the complex manipulation of electronic devices. They examined the stress effects on eye movements in responses obtained from two evaluation experiments. The first experiment targeted transient stressors. They set a time constraint for participants to respond to images that were displayed randomly in the four corners of a monitor. They examined the correspondence between saccades and two emotions, including frustration (with a long time limit) and impatience (with a short time limit). Although task-relevant saccades of wide amplitude were uncorrelated with these feelings, task-irrelevant saccades of narrow amplitude showed a positive correlation. The second experiment targeted perceptual stressors, demonstrating that subjects responded to the object names from images of different quality and modalities. Stress was assessed quantitatively from indexes of impatience, confusion, and activity levels for visual tasks that induced degrees of progressive stress from reduced visibility in addition to operational difficulties. However, in all tasks, no correlation was shown between stress and saccades.

Iizuka et al. [72] specifically examined the relation between gaze and emotions in human communication. They analyzed not only the factors and intensity of positive and negative emotions but also the profiles of communication partners. After memorizing two emotions of sentences given to them in advance, each including pleasant and unpleasant expressions, the participants expressed the sentences according to the context. The experimentally obtained results demonstrated that gaze areas and saccades increased for a female communication partner. By contrast, it is reduced in a male communication partner while expressing negative emotions.

Our earlier study [23] specifically examined stress responses of participants who had earlier watched emotion-provoking videos as pleasant and unpleasant stimuli. We examined the responses, which indicated the effectiveness of these videos as a transient stressor. We obtained an original dataset consisting of hemoglobin (Hb) based on cerebral blood flow patterns obtained from a portable near-infrared spectroscopy (NIRS) device, HR, and salivary amylase as a biological index, self-evaluation scores of five levels as a psychological index, and gaze and saccades as a behavioral index. Particularly, we attempted to quantify the relation between Hb and stressors. A comparison of the results obtained for Hb differences demonstrated that the respective videos were effective for transient stressors. Moreover, gaze distribution in the section of wide Hb changes demonstrated concentrations for positive stimulus and dispersion for negative stimulus. We concluded that saccades are useful for a stress index, and gaze areas are useful for a positive emotional index.

### 2.2. Facial Expressions

Facial expressions [73] provide diverse information. Automatic analysis of facial expressions is a highly challenging task in computer vision studies [74]. Typically, intermediate facial expressions include several face parts in parallel with several emotions, such as a smiling mouth and sad eyes [75]. Similar to the differences in face shapes for each person, expression patterns and their speed include individual differences such as expression ranges of facial changes for a particular emotion. Moreover, we sense rhythms not only from conversations but also from various surroundings in our daily lives, such as moving targets and sound sources. Our earlier study [76] defined a personal tempo as the time-series feature combination of facial expression changes. As a conceptual definition, the personal tempo represents individual behavior pattern speeds that occur naturally for free motions, with no restrictions on our daily behavior patterns such as speaking, walking, and sleeping. Particularly, we considered that facial expressions include individual rhythms and tempos because facial expressions appear not only unconsciously when triggered by emotions but also consciously when triggered by desires to make a positive impression for social communication.

We defined a facial expression tempo [76] as a distinct part among expressionless points via a particular expression, as measured by facial expression spatial charts (FESC) [77]. Moreover, we defined a facial expression rhythm as the time-series feature combination of tempos for each person, reflecting their individual habits of communication. Using these frameworks and emotion-provoking videos, we examined the effects of pleasant and unpleasant stimuli on facial expressions. We attained the number of frames that comprise a tempo of expression changes for stimulus and its fluctuations from transient stressors. Moreover, we specified facial expressions and face parts that exhibit stress effects. The degree of mutual information related to tempos and rhythms in facial parts suggested the possibility of estimating impressions given by facial expressions [78]. We consider that this framework is useful for the measurement of the naturalness or unnaturalness of facial expressions.

As an analytical study of the relation between facial expressions and mood states, Hamada et al. [79] emphasized the eyes, eyebrows, mouth, and body movements between facial expressions and an electroencephalogram (EEG) as a physiological index. They examined the relation mood states and features of each face part using EEG. The experimentally obtained results demonstrated that α waves, which appear in a relaxed and pleasant state, were dominant in the case of smaller eye width, lower eyebrow position, and greater mouth opening length. In this case, the body moved left and right with natural body movements. Moreover, the experimentally obtained results demonstrated that θ waves, which appear in a concentrated state, were dominant in the case of wider opening eyes, upper eyebrow positions, and a greater mouth opening length. In this case, the body tended to move up and down intentionally.

Arita et al. [80] proposed a method of estimating dominant emotions using four indicators: HR, facial expression patterns, facial surface temperature, and pupil diameter. They developed an original benchmark dataset using emotion-provoking videos. The experimentally obtained results showed correlations with three other indices associated with facial expression changes: the face temperature decreased by approximately 1 °C to the temperature of the nasal region during the presentation of deep images; the HR showed an increase in HR frequency upon presentation of the unpleasant video; and the pupil diameter increased concomitantly with increasing arousal levels. Correlations between these measurements and the membership scores of the subjective ratings were assessed using canonical correlation analysis. Although the experimentally obtained emotion discrimination accuracy was 40–56%, response patterns differed widely among subjects. They considered that one major reason for this result derived from a tendency by which subjects were hesitant to express their emotions.

Ueda et al. [81] examined the effects of individual differences in neutral facial expressions to estimate impressions with a communication partner. They conducted evaluation experiments to recognize impressions of facial expressions based on subjective evaluation indexes for pleasant and unpleasant feelings from viewing photographs with a smile and neutral expressions. Their experimentally obtained results revealed that differences in static expressions that were specific for individuals had a consistent effect on impressions during the viewing of the expressions.

As described above, research investigating stress varies enormously, not only in its approach and sensing methods but also in its measurement targets and evaluation criteria. For this study, we examine changes in facial expressions, especially during repeated intentional smiles. We define one tempo as a cycle from an expressionless condition to another expressionless condition via a smile expression. We obtained time-series images including intermediate, affectionate, and natural smiles. Classification and extraction of natural facial expression patterns in human communication are expected to lead to the elucidation of the relation between stress and mood states.

## 3. Dataset

### 3.1. Experiment Environment

**Figure 2:**
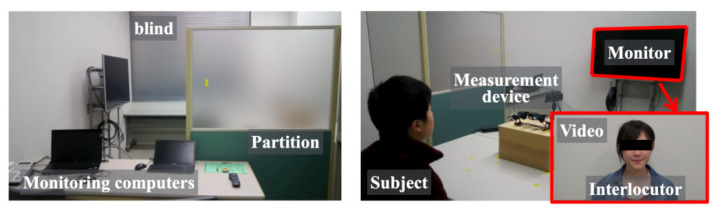
The experiment room is divided into two sections for a subject and an experimenter (**Left**). The subject watches the monitor, which is placed at a 3 m distance across the table (**Right**). A video showing communication with an interlocutor is presented on the monitor.

Figure 2 depicts the room used for an experiment environment to obtain benchmark datasets. The partition installed in the room of 20 m${}^{2}$ separated the sections for a subject and an experimenter as an observer. The laptop computers on the desk at the front side of the room were connected to a measurement device for data collection. The experimenter monitored the progress of experimental protocols and the responses of subjects. After sitting on a chair at the back of the room, the subject watched the 50-inch monitor placed at 3 m distance across the table. The facial measurement device was set up on the table. A video for communication with an interlocutor was shown on the monitor. We took care to maintain silence in the room to allow subjects to undergo the experiment in a relaxed condition. The blinds on the windows were closed for protection from sunlight. The room temperature and humidity were kept constant using an air-conditioner.

### 3.2. Sensing Device

**Figure 3:**
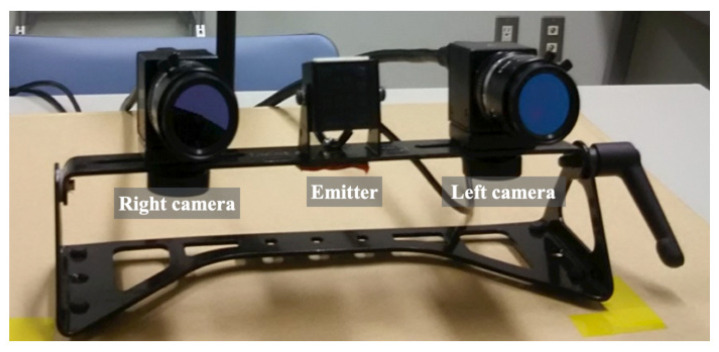
Exterior of the faceLAB 5 head-and-eye tracking device.

For gaze tracking and saccade extraction, we used faceLAB 5 (Seeing Machines Inc.; Fyshwick, ACT, Australia), as depicted in Figure 3. The faceLAB 5 apparatus comprises an emitter and a stereo camera with 0.5–1.0 deg angular resolution and 60 Hz data sampling. The included application software produces heatmap results calculated from gaze concentration density and the number of saccades, which are defined as rapid eye movements between fixation points.

### 3.3. POMS2

We used the Profile of Mood States Second edition (POMS2) [82] sheets for measuring psychological information from the respective subjects. POMS2 is used at clinical sites such as those for medical care, nursing, welfare, and counseling.

POMS2 consists of seven mood components: anger–hostility (AH), confusion–bewilderment (CB), depression–dejection (DD), fatigue–inertia (FI), tension–anxiety (TA), vigor–activity (VA), and friendliness (*F*). For each component, subjects give responses according to five-point scales. The total mood distance (TMD) is calculated as

(1) TMD=AH+CB+DD+FI+TA−VA,

where VA is inverted. Component *F* is an index that is independent of TMD. For clustering mood states, we define TMD as the primary component and *F* as the secondary component.

With the different item numbers, POMS2 provides three versions. The numbers of items for a youth version for young people between 13 and 17 years old, an adult version for more than 18 years old, and a simplified adult version are, respectively, 60, 65, and 35 items. The subjects for this experiment were all university students older than 18 years old. They were applicable to the adult version of POMS2 in Japanese [83]. Regarding the total experiment time, we used the simplified version. The mean answer time was approximately five minutes.

After standardizing the prime scores, we calculated T-scores with a mean of 50 and a standard deviation of 10. The T-score conversion normalizes the metrics of assessment in terms of numerical equivalents. This normalization provides the possibility of appropriate comparison among individual examinations for the obtained scores, scales, and forms.

### 3.4. Obtained Datasets

Our original benchmark dataset was obtained from 20 university students, 10 male and 10 female, through volunteer sampling. Table 1 comprises the profiles of respective subjects. The data collection interval was set to one week to reduce the effects of the response for a frequently repeated stimulus. Regarding restrictions for subjects, the total measurement terms were set to two types: four weeks for 10 subjects and eight weeks for 10 subjects. Therefore, the total data volume is 120 sets.

## 4. Proposed Method

**Figure 4:**
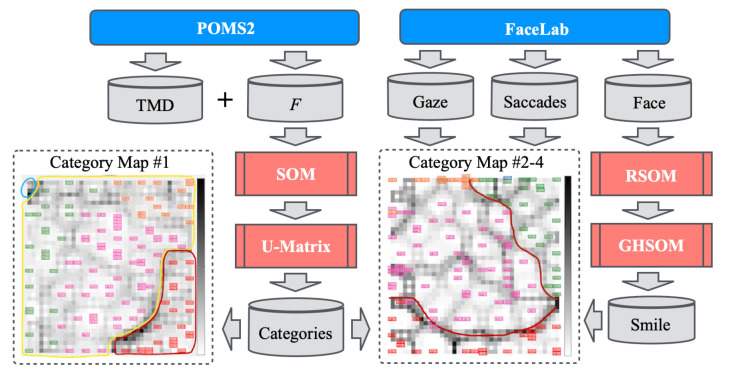
Structure and data flow of our proposed method. Category maps are created from several combinations of input features.

Our original benchmark dataset includes no ground truth (GT) labels. Therefore, we employed unsupervised learning methods. Figure 4 depicts the entire procedure of our proposed method, comprising four ML algorithms: SOM [26], recurrent SOM (RSOM) [84], growing hierarchical SOM (GHSOM) [85], and U-Matrix [86]. The TMD and *F* scores, gaze features, saccades, and face images were obtained from POMS2, FaceLab, and a monocular camera. First, categories related to mood status are created using SOM and U-Matrix from TMD and *F*. Gaze features and saccades are used for analyzing the obtained categories. Subsequently, smile images are extracted using RSOM and GHSOM from time-series face images obtained from a monocular camera.

**Figure 5:**
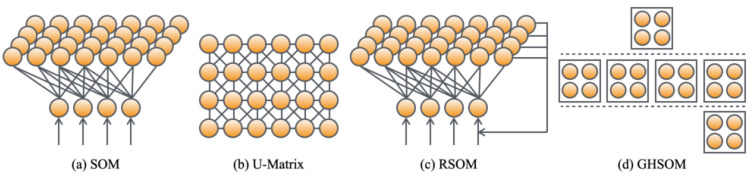
Network structures of SOM, U-Matrix, RSOM, and GHSOM. SOM and U-Matrix are used for creating category maps. RSOM and GHSOM are used for extracting smile-expressed frames.

Figure 5 depicts the respective network structures. The core algorithms of U-Matrix, RSOM, and GHSOM were designed based on SOM.

### 4.1. SOM

Letting $x_{i}\left(t\right)$ denote the features to input layer unit *i* at time *t*. Furthermore, letting $w_{ijk}\left(t\right)$ denote a weight from *i* to mapping layer unit (j,k) at time *t*. Before learning, values of $w_{ijk}\left(t\right)$ are initialized randomly. Using the Euclidean distance between $x_{i}\left(t\right)$ and $w_{ijk}\left(t\right)$, a winner unit $c_{j}\left(t\right)$ is sought for the following as

(2) $$c_{j}\left(t\right)=\underset{1\leq j\leq J,1\leq k\leq K}{\mathrm{argmin}}\sqrt{\sum_{i=1}^{I}{\left(x_{i}\left(t\right)-w_{ijk}\left(t\right)\right)}^{2}},$$

where *I* and (J,K), respectively, denote the total numbers of input layer units and mapping layer units.

A neighboring region ψ(t) is set from the center of $c_{j}$ as

(3) $$\psi \left(t\right)=\lfloor \psi \left(0\right)\cdot J\cdot \left(1-\frac{t}{O}\right)+0.5\rfloor ,$$

where *O* represents the maximum of learning iterations. Subsequently, $w_{ijk}\left(t\right)$ in ψ(t) is updated as

(4) $$w_{ijk}\left(t+1\right)=w_{ijk}\left(t\right)+\alpha \left(x_{i}\left(t\right)-w_{ijk}\left(t\right)\right),$$

where α is a learning coefficient that decreases according to the learning progress. Herein, at time t=0, we initialized $w_{ijk}\left(0\right)$ with random numbers.

### 4.2. U-Matrix

U-Matrix [86] is used for extracting cluster boundaries from $w_{ijk}$. Based on metric distances between weights, U-Matrix visualizes the spatial distribution of categories from the similarity of neighbor units [86]. On a two-dimensional (2D) category map of square grids, a unit has eight neighbor units, except for boundary units. Letting *U* denote the similarity calculated using U-Matrix. For the component of the horizontal and vertical directions, $U_{h\pm }$ and $U_{v\pm }$ are defined as shown below.

(5) $$U_{h\pm }=\sqrt{\sum_{i=1}^{I}{\left(w_{ijk}-w_{ij\pm 1k}\right)}^{2}},U_{v\pm }=\sqrt{\sum_{i=1}^{I}{\left(w_{ijk}-w_{ijk\pm 1}\right)}^{2}}.$$

For components of the diagonal directions, $U_{d\pm }$ are defined as presented below.

(6) $$U_{d\pm }=\frac{1}{2}\sqrt{\sum_{i=1}^{I}{\left(w_{ijk\pm 1}-w_{ij\pm 1k}\right)}^{2}}+\frac{1}{2}\sqrt{\sum_{1=1}^{I}{\left(w_{ij\pm 1k}-w_{ijk\pm 1}\right)}^{2}}$$

### 4.3. Recurrent SOM

Our method uses RSOM for extracting smile images from time-series facial expression images [87]. As a derivative model of SOM [26], RSOM [84] incorporates an additional feedback loop for learning time-series features. Temporally changed input signals are mapped into units on the competitive layer. $\beta_{1}$, $\beta_{2}$, $\beta_{3}$ were set as denoting learning coefficients. The output $y_{jk}\left(t\right)$ from the mapping unit (*j*, *k*) at time *t* is presented as the following.

(7) $$\begin{array}{c} y_{jk}\left(t\right)=\beta_{1}y_{jk}\left(t\right)+\beta_{2}y_{jk}\left(t-2\right)+\beta_{3}\left(x_{i}\left(t\right)-w_{ijk}\left(t\right)\right). \end{array}$$

The weights are updated as

(8) $$\begin{array}{c} w_{ijk}\left(t+1\right)=\left(1-\gamma \right)w_{ijk}\left(t\right)+\gamma \left(x\left(t\right)-w_{ijk}\left(t\right)\right)y_{jk}\left(t\right), \end{array}$$

where γ is a learning coefficient that decreases according to the learning progress.

The RSOM mapping size is set in advance with the number of units. This parameter controls the classification granularity of facial expression images. This method affixed 15 units based on the setting parameter of FESC in our earlier study [77].

### 4.4. GHSOM

As an extended SOM network and its training algorithm, GHSOM [85] incorporates a hierarchization mechanism that accommodates an increased number of mapping layers. An appropriate mapping size for solving a target problem is obtainable automatically by GHSOM. Although the weight update mechanism of GHSOM resembles that of SOM, the learning algorithm of GHSOM includes the generation of a hierarchical structure based on growing and adding mapping units in each layer, except the top layer. The respective GHSOM layers provide parallel learning as independent modules.

The growing hierarchical algorithm is launched from the top layer, which comprises a single unit [88]. Letting $w_{0}$ denote a weight between the top layer and the next layer. The top layer, which includes no growing mechanism, branches into four sub-layers. All sublayers have 2 × 2 mapping units. Growing hierarchical learning is actualized on the units of the sublayers. Letting $v_{i}$ denote a standard deviation for the input $x_{i}$ to the mapping units of the *i*-th sub-layer. The mean standard deviation $v_{m}$ is calculated as presented below.

(9) $$\begin{array}{c} v_{m}=\frac{1}{I}\sqrt{\sum_{i=0}^{I}\sum_{j=0}^{J}\sum_{k=0}^{K}\left(w_{ijk}-x_{i}\right)}. \end{array}$$

Letting $T_{m}$ represent the breadth threshold. Hierarchical growing is controlled by $T_{m}$, as presented below.

(10) $$\begin{array}{c} T_{m}<\frac{v_{m}}{v_{m-1}}. \end{array}$$

A unit for growing is appended if the ratio between $v_{m}$ of the *m*-th layer and $v_{m-1}$ of the (m−1)th layer is greater than $T_{m}$.

The hierarchical growing procedure for adding new units comprises four steps. The first step is the specification of an error unit $u_{e}$ that indicates the maximum standard deviation between units. The second step is the selection of a dissimilar unit $u_{d}$ that indicates a minimum standard deviation from neighboring units around $u_{e}$. The third step is the insertion of a new unit between $u_{e}$ and $u_{d}$. The fourth step is updating of weights of the respective units based on the SOM learning algorithm. After learning, input features are classified again. The standard deviation is decreasing according to the growing progress. The growth termination is triggered by saturation of the added units as a suitable mapping size. After adding units, the addition of new layers is processed. Finally, the learning phase is completed if growing is terminated.

### 4.5. Parameters

Table 2 denotes the meta-parameters of SOM, RSOM, and GHSOM and their initial setting values. We set them based on our earlier study [23]. The parameter *I* is changed according to the input dimensions in each experiment.

## 5. Experiment Results

### 5.1. Unsupervised Classification Results of Mood States

**Figure 6:**
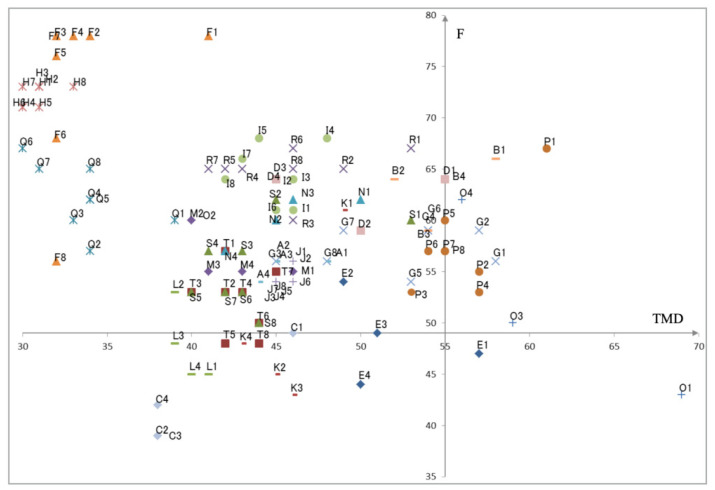
The 2D-distribution of TMD and *F* calculated from POMS2 T-scores for all subjects.

Figure 6 presents the TMD distribution on the horizontal axis and *F* scores on the vertical axis. These scores are calculated from POMS2 T-scores. They can therefore be 120 plots from the dataset denoted in Table 1. The intersection of the axes corresponds to the mean scores of TMD and *F* obtained from [89]. On the one hand, small and large TMD scores can be interpreted, respectively, as positive and negative mental states. On the other hand, small and large *F* scores can be interpreted, respectively, as negative and positive mental states.

**Figure 7:**
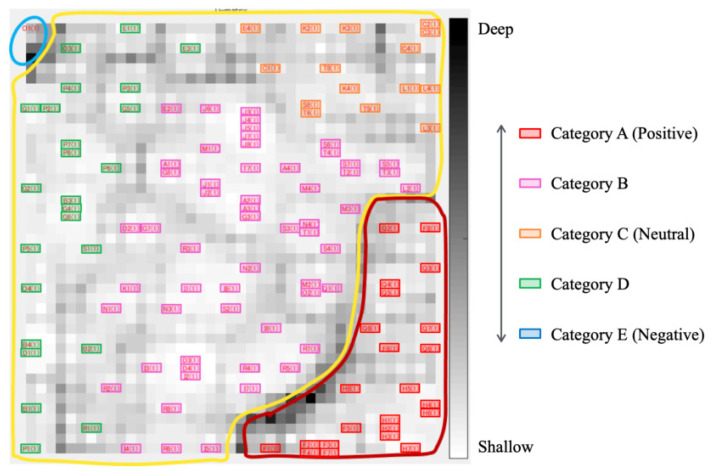
Category boundary and unsupervised classification results with U-matrix from the input of TMD and *F*.

Based on unsupervised clustering of the data plots, a category map was created with SOM. Figure 7 shows the result with categorical boundaries extracted from U-Matrix. The brightness represents the depth of categorical boundaries. Lower and higher brightness scale values, respectively, indicate deeper and shallower boundaries. Deeper category boundaries appeared in the upper-left and bottom-right areas on the map. These boundaries divided the category map into three independent regions. Moreover, three categories were extracted from the left half, upper right half, and the bottom right half in the category enclosed by the solid yellow border. Regarding the relation between this classification result and the distribution in Figure 6, five categories labeled Categories A–E were obtained from Figure 7.

**Figure 8:**
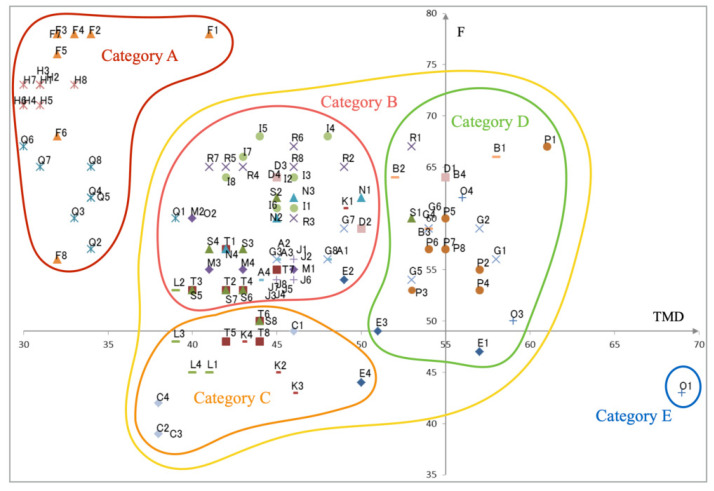
Unsupervised classification results for the data in Figure 6 based on Figure 7.

Figure 8 depicts a classification result of the coordinate points in Figure 6 based on the five categories extracted from Figure 7. Category A is defined semantically as positive, which is attributable to their low TMD and high *F* scores. By contrast, Category E is defined semantically as negative, which is attributable to their high TMD and low *F* scores. Based on the positional relations, the three categories distributed around the center are defined, respectively, as positive for Category B, negative for Category D, and neutral for Category C. Fundamentally, we affixed these semantic labels based on the vertical axis associated with TMD scores. The decision boundary is located around 51 points, which is lower than the mean score of 55 points. We referred to *F* scores to affix semantic labels for Categories B and C, which are located in similar TMD ranges. The decision boundary is located near 52 points, which is higher than the mean score of 49 points. The data shown for respective subjects, as presented in Table 1, include intra-categorical and inter-categorical distribution patterns. For subjects with widely diverse mood states, the data plots on the horizontal axis tend to be long. By contrast, for subjects with a narrow range of mood states, data plots on the horizontal axis tend to be short. Based on these parameters, we analyzed the relation between representative subject data obtained from POMS2 and the eye-tracking device.

### 5.2. Relation between Mood State and Gaze Distribution

**Figure 9:**
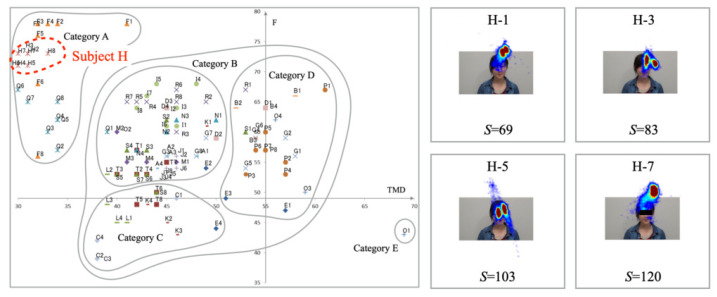
Mood states (**Left**) and gaze distribution (**Right**) for Subject H in Category A.

This evaluation experiment yielded representative results obtained from analyzing the relation between mood states and gaze distribution features, including saccades for six subjects: Subjects H, F, I, C, B, and O, in that order. Figure 9 presents the results obtained for Subject H. The mode states plots are distributed inside Category A. The heatmap results show the gaze distribution and its density gathered around the interlocutor’s face on the monitor. The tendency with small changes represents the steady mood states and gaze distribution to specific areas. The number of saccades is smaller than those of other subjects.

**Figure 10:**
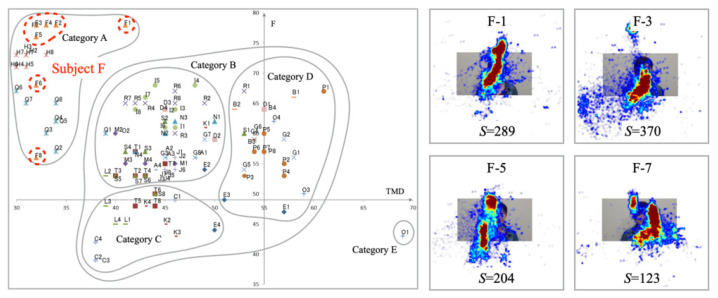
Mood states (**Left**) and gaze distribution (**Right**) for Subject F in Category A.

Figure 10 presents experiment results for Subject F. Unlike Subject H, the plots of mood states are distributed over the whole of Category A. The distribution range of *F* is greater than that of TMD. The experimentally obtained results demonstrate not only wider gaze distribution but also a greater number of saccades compared with those of Subject H.

**Figure 11:**
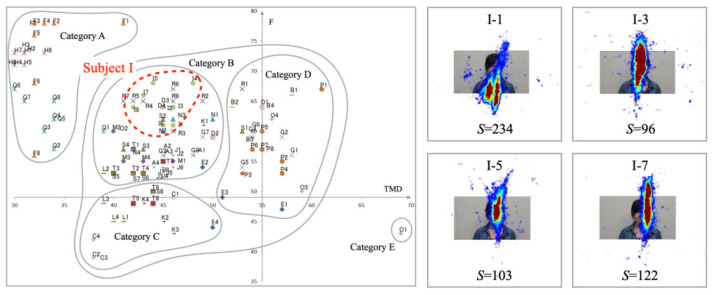
Mood states (**Left**) and gaze distribution (**Right**) for Subject I in Category B.

Figure 11 presents experimentally obtained results for Subject I. The plots of mood states are distributed in the upper half of Category B. Although the gaze was concentrated on the interlocutor’s face on the monitor, the distribution shape was spread vertically. A high number of saccades indicates severe vertical movements of the eyes.

**Figure 12:**
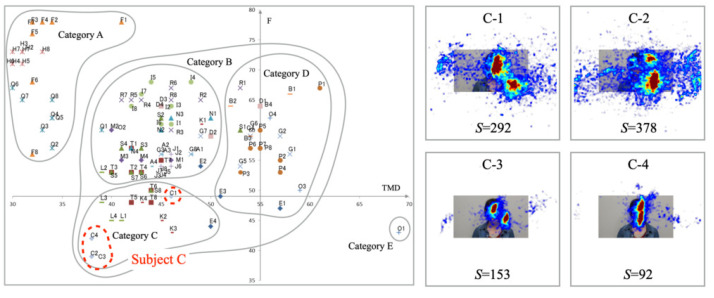
Mood states (**left**) saccade distributions (**right**) for Subject C in Category C.

Figure 12 portrays experimentally obtained results for Subject C. The plots of mood states are distributed in two parts in Category C. The gaze distribution was unstable, with two of the four cases extending their range laterally. The heatmap results demonstrate that the gaze distribution of three of the four cases is divided into two clusters. The number of saccades increased with the expansion of the gaze area. Although the mood states differed from those of the other three samples in the distribution of C-1, no characteristic association with gaze was identified.

**Figure 13:**
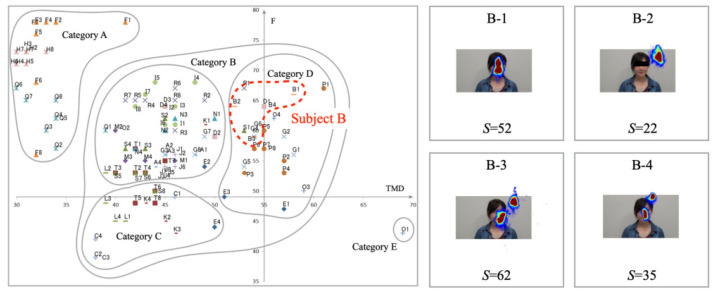
Mood states (**left**) saccade distributions (**right**) for Subject B in Category D.

Figure 13 depicts experiment results for Subject B. The distribution of the mood states is placed in Category D. The range of TMD and *F* suggests that the changes in mood states for this subject are narrow. The high-temperature heatmap results indicate that the gaze plots are gathered densely to the interlocutor’s face on the monitor. The number of saccades is smaller than for the other subjects. We consider that gaze movements are steady for questioners that are attributable to a lack of mood state changes.

**Figure 14:**
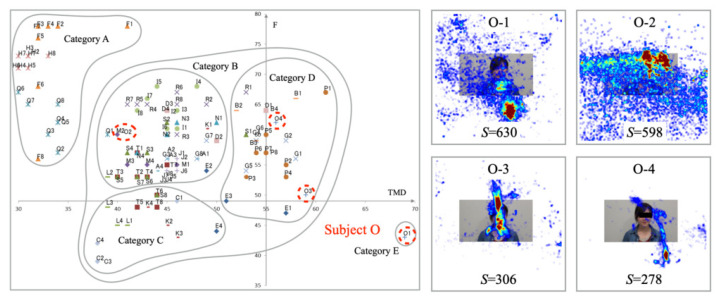
Mood states (**left**) saccade distributions (**right**) for Subject O in Categories B, D, and E.

Figure 14 portrays the results obtained for Subject O. The mood states are distributed in three categories: Categories B, D, and E. Although the gaze plots are distributed widely, their concentrating area is narrow. High-temperature areas on the heatmap are gathered for the interlocutor, except for the O-3 example. We infer that gaze distribution patterns affected the mood states of this subject. The number of saccades tended to increase with wide eye movements and decrease with narrow eye movements.

### 5.3. Smile Expression Extraction

Face regions were extracted using the Viola-Jones [90] method, which is a dominant object detection framework based on Haar-like features combined with Ada-boost cascading classifiers. Regarding the camera position, view angle, and resolutions, we extracted a fixed region of interest (RoI) of 320×320 pixels. The final purpose of this experiment is to visualize mental health displayed on a 2D map created from several feature combinations.

**Figure 15:**
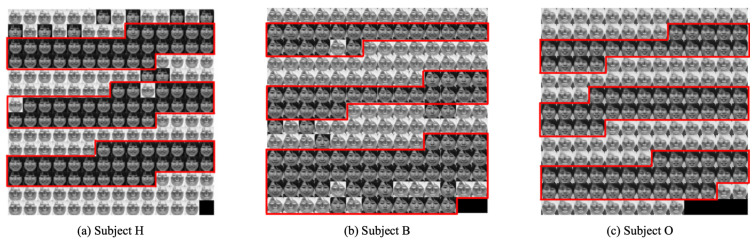
Extraction result of smile expression images for three subjects: Subjects H, B, and O.

The RSOM module extracted smile-expressed frames from time-series facial images. Figure 15 presents extraction results for the representative three subjects: Subjects H, B, and O. Positive images and negative images, respectively, correspond to the smile expressions and blank expressions. The red frames show GT images labeled as smile expressions. Although mismatched images exist among the frames of low expression intensity, our method globally extracted smile images concomitantly with the GT frames.

For annotators, classifying images that switch facial expressions is a difficult task. Moreover, annotating the facial expression images of women is more difficult than annotating those of men. The accuracy of extracting smile images with RSOM demonstrated that over 90% was similar to the accuracy obtained from our earlier study. The odd frames and even frames were set, respectively, to training and validation subsets. Gabor wavelets transformations [91] were applied to input data images of 160×160 pixels. Based on FESC [77], 15 weights corresponding to 15 units obtained from RSOM were attended to GHSOM.

**Figure 16:**
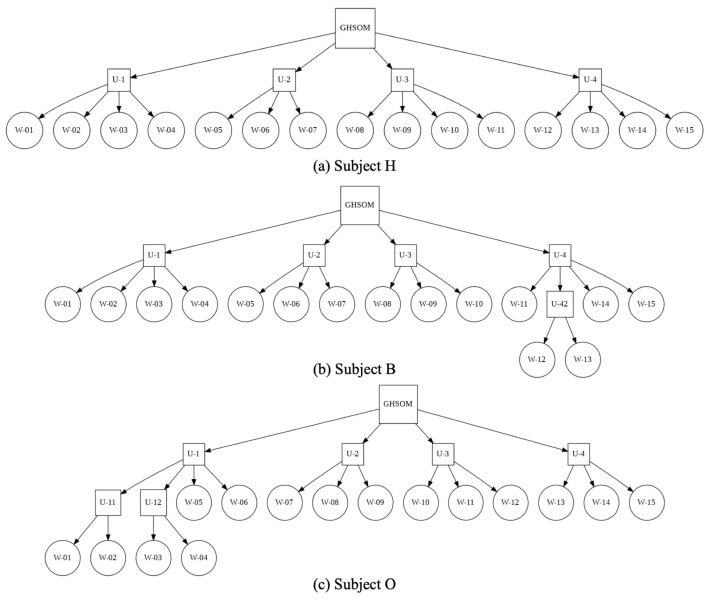
Hierarchical unsupervised classification results with RSOM weights with GHSOM for three subjects: H, B, and O.

Smile images were classified hierarchically using GHSOM using RSOM weights. Figure 16 depicts unsupervised classification results, presented as tree structures for Subjects H, B, and O. The RSOM mapping layer size was set to 15 units, which divides weight into 15 clusters. The maximum granularity was set to four categories in each layer. The weights of Subject H were divided into four clusters in the first depth layer. The weights of Subject B were divided into five clusters. In the fourth cluster, two weights were categorized in the second depth layer. The weights of Subject O were divided into six clusters. In the first cluster, four weights were categorized into two clusters in the second depth layer.

### 5.4. Effects of Input Features on Visualization Results

This experiment was conducted to verify the relation between gaze patterns and facial expressions that affect changes in mood states. For experimentation, we used the visualization modules based on SOM and U-Matrix. We set *E*, *S*, *R*, and *G* as, respectively, denoting the number of pixels extracted from gaze movements, the number of saccades, the number of smile images obtained from RSOM, and the number of categories obtained from GHSOM. This experiment provides three input patterns based on TMD and *F* combined with *E*, *S*, *R*, and *G*, as presented below.

- Input I: TMD +F+E+S
- Input II: TMD +F+R+G
- Input III: TMD +F+E+S+R+G

**Figure 17:**
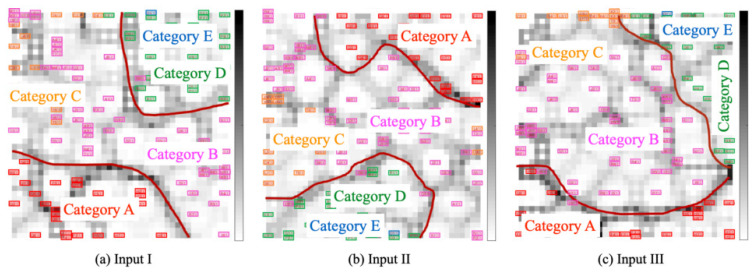
Unsupervised classification results obtained for three input patterns. Red curves are drawn subjectively as category boundaries.

The combination of these input features provides different distribution patterns on category maps as visualization results. Figure 17a presents unsupervised classification results for Input I. Annotation labels, which correspond to the categories in Figure 8, are superimposed on the category map. Categories A and D were allocated, respectively, to the bottom left and upper right on the map. Categorical boundaries appeared to be deeper and more continuous than the result obtained for the input of TMD and *F* in Figure 7. Unsupervised classification results for Input I demonstrated that the gaze-related features are useful to delineate the boundaries of the categories, especially in Category A. Figure 17b depicts the classification result obtained for Input II. Categories A and D were allocated, respectively, to the bottom left and upper right on the map. Figure 17c depicts unsupervised classification results obtained for Input III. Categories A and D were allocated, respectively, to the bottom and upper right on the map. Categorical boundaries appeared to be deeper and more continuous than the results obtained for Inputs I and II.

## 6. Conclusions

This paper presented a method of classification and visualization of mood states obtained from a psychological check sheet and facial features of gaze, saccades, and facial expressions based on unsupervised ML algorithms. The two indicators TMD and *F* obtained from POMS2 were classified into five categories using SOM and U-Matrix. Relations between gaze and facial expressions were analyzed from the five extracted categories. Subjects in positive categories demonstrated positive correlations between gaze concentration areas colored with high-temperature heatmaps and the number of saccades. In particular, for subjects with widely diverse mood states, the gaze data distributions on the horizontal axis tend to be long. By contrast, for subjects with a narrow range of mood states, gaze data distributions on the horizontal axis tend to be short. Regarding facial expressions, positive category subjects had a constant expression time of intentional smiles. By contrast, subjects in the negative categories exhibited a time length difference in intentional smiles. Furthermore, we examined the influences of gaze and facial expressions on category classification using RSOM and GHSOM. The results obtained from three comparative experiments indicated that adding features of gaze and facial expression to TMD and *F* clarified the category boundaries obtained from the U-Matrix. Compared to the result obtained for the input of TMD and *F*, categorical boundaries appeared to be deeper and more continuous using features of the number of pixels extracted from gaze movements, the number of saccades, the number of smile images obtained from RSOM, and the number of categories obtained from GHSOM. We verify that the use of SOM, RSOM, and GHSOM is the best combination for the visualization of mood states.

In future work, we would like to actualize stress estimation only from gaze or facial expressions. The only subjects for this experiment were university students in their 20s. We would like to expand the application range of the proposed method, especially for a wider age range of subjects. Moreover, we plan to develop apps for tablet computers and smartphones to facilitate the practical application of this method.

## Figures and Tables

**Table 1:** Profiles and measurement terms for subjects.

Subject	Sex	Age	Number of Measurements	Label
A	Male	22	4	A1–4
B	Female	22	4	B1–4
C	Male	23	4	C1–4
D	Female	22	4	D1–4
E	Male	22	4	E1–4
F	Female	21	8	F1–8
G	Male	22	8	G1–8
H	Female	22	8	H1–8
I	Male	24	8	I1–8
J	Female	22	8	J1–8
K	Male	22	4	K1–4
L	Female	22	4	L1–4
M	Male	21	4	M1–4
N	Female	22	4	N1–4
O	Male	22	4	O1–4
P	Female	22	8	P1–8
Q	Male	20	8	Q1–8
R	Female	22	8	R1–8
S	Male	23	8	S1–8
T	Female	22	8	T1–8

**Table 2:** Meta-parameters and initial setting values.

Method	Parameter	Description	Default
SOM	$w_{ijk}\left(t\right)$	weights	random
	*I*	number of input units	2
	*J*	number of vertical mapping units	50
	*K*	number of vertical mapping units	50
	ψ(0)	number of neighborhood region	40
	α	learning coefficient	0.1
	*O*	maximum learning epochs	200
RSOM	$w_{ijk}\left(t\right)$	weights	random
	$\beta_{1}$	learning coefficient	0.5
	$\beta_{2}$	learning coefficient	0.2
	$\beta_{3}$	learning coefficient	0.3
	γ	learning coefficient	0.1
	*O*	maximum learning epochs	200
GHSOM	$w_{ijk}\left(t\right)$	weights	random
	$T_{m}$	breadth threshold	0.08
	*O*	maximum learning epochs	200

## Data Availability

Datasets presented as a result of this study are available on request to the corresponding author.

## Abbreviations

The following abbreviations are used in this report:

2D	Two-dimensional
DL	Deep learning
EEG	Electroencephalogram
FESC	Facial expression spatial charts
GHSOM	Growing hierarchical self-organizing maps
GT	Ground truth
Hb	Hemoglobin
HR	Heart rate
ML	Machine-learning
NIRS	Near-infrared spectroscopy
POMS2	Profile of mood states second edition
RSOM	Recurrent self-organizing maps
RoI	Region of interest
SNS	Social networking services
SOM	Self-organizing maps
TMD	Total mood distance
